# Citrus transcription factor CsERF1 is involved in the response to citrus tristeza disease

**DOI:** 10.3389/fpls.2024.1528348

**Published:** 2025-01-14

**Authors:** Qi Chen, Fulin Yan, Jing Liu, Zhipeng Xie, Junyao Jiang, Jiamei Liang, Jing Chen, Huanhuan Wang, Jinxiang Liu

**Affiliations:** National Citrus Engineering and Technology Research Center, Citrus Research Institute, Southwest University/Integrative Science Center of Germplasm Creation in Western China (CHONGQING) Science City, Citrus Research Institute, Southwest University, Chongqing, China

**Keywords:** citrus, citrus tristeza virus, CsERF1, salicylic acid, jasmonic acid

## Abstract

**Introduction:**

Citrus tristeza virus (CTV) is a threat to the citrus production and causes severe economic losses to the citrus industry. Ethylene response factors (ERFs) play important roles in plant growth and stress responses. Although ERF genes have been widely studied in model plants, little is known about their role in biological stress responses in fruit trees, such as citrus. CsERF1 belongs to the citrus AP2/ERF transcription factor family.

**Methods:**

To determine the role of CsERF1 on CTV resistance in citrus and the effects of the exongenous hormone application on CsERF1 in citrus, the expression of related genes was quantitatively analyzed by quantitative reverse transcription polymerase chain reaction (RT-qPCR) in this study.

**Results:**

The expression profile showed that the expression level of *CsERF1* in roots was significantly lower under CTV infection than in healthy plants, while the expression level in stems was significantly increased. *CsERF1* responded to exogenous salicylic acid (SA) and methyl jasmonate (MeJA) treatments. The CTV titer in RNAi-CsERF1 transgenic sweet orange plants significantly increased. Furthermore, *CsERF1*-overexpressing and RNAi-CsERF1 transgenic sweet orange plants exhibited differential expression of genes involved in jasmonic acid (JA) and SA signaling.

**Discussion:**

These results suggest that CsERF1 mediates CTV resistance by regulating the JA and SA signaling pathways. The results of this study provide new clues as to the citrus defence response against CTV. It is of great significance to create citrus germplasm resources resistant to recession disease.

## 1 Introduction

Citrus is one of the major fruit tree crops throughout the world. However, citrus production is limited by disease. Tristeza, which causes great economic losses in the citrus industry, is caused by citrus tristeza virus (CTV), a member of the genus *Closterovirus* in the family Closteroviridae. During the last century, severe epidemics of quick decline caused by CTV have killed almost 100 million citrus trees grown on sour orange rootstock worldwide (Catara et al., 2021). Citrus plants infected with CTV show developmental retardation, seedling yellowing, wilting, stem pitting, and small fruit, which affect citrus quality and yield (Dawson et al., 2015; Garnsey et al., 2005). Presently, the only possibility to protect susceptible commercial varieties from virulent CTV isolates is classical cross protection with mild CTV isolates, but the effectiveness of protection was influenced by regional condition and host factors (Folimonova et al., 2020). Thus, investigating the pathogenesis of CTV is essential for generating CTV-resistant citrus germplasm in citrus cultivar breeding.

Members of the ethylene response factor (ERF) subfamily, a branch of the APETALA2/ETHYLENE RESPONSIVE FACTOR (AP2/ERF) superfamily, play important roles in plant morphogenesis, stress response mechanisms, and metabolite regulation (Feng et al., 2020; Yin et al., 2021). Furthermore, studies have indicated that ERF transcription factors specifically bind to the GCC element (AGCCGCC) to activate or suppress the transcription of target genes (Fujimoto et al., 2000; Liang et al., 2008) involved in pathogen infection and disease resistance in plants. For example, *OsBIERF3* overexpression enhances disease resistance and salt tolerance in transgenic tobacco, while increased expression levels of the defense-related *PR-1a* gene have also been detected (Cao et al., 2005). *AtERF4* and *AtERF14* in *Arabidopsis thaliana* play a key role in plant resistance responses to the necrotrophic fungal pathogen *Fusarium oxysporum* (McGrath et al., 2005; Oñate-Sánche et al., 2007). *TSRF1* overexpression in tobacco and tomato enhances the resistance of transgenic plants to *Ralstonia solanacearum* (Zhang et al., 2004). In contrast, *AtERF19* overexpression increased plant susceptibility to *Botrytis cinerea* and *Pseudomonas syringae* and inhibited pathogen-associated molecular pattern (PAMP)-triggered immunity (PTI) (Huang et al., 2019). *OsERF922*-overexpressing rice plants showed reduced expression of defense-related genes and enhanced susceptibility to *Magnaporthe oryzae* (Liu et al., 2012). The function of ERF genes in response to the pathogen differs, depending on the gene, pathogen, and host plant. Although AP2/ERF genes have been widely studied in model plants, such as *Arabidopsis thaliana*, little is known about their role in biological stress responses in perennial fruit crops, such as citrus.

ERF transcription factors are also an important regulatory site of the plant hormone defense signal crossing pathway, participating in salicylic acid (SA), jasmonic acid (JA), and other hormone signal defense pathways. For example, *SlERF01* has been shown to enhance tomato disease resistance through SA and JA defense pathways. *SlERF01* of the tomato ERF family is induced by exogenous SA and JA application, which activate the expression of the *PR1* gene and enhance resistance to *Stemphylum lycopersici* (Yang et al., 2020). MeJA treatment induces the expression of *NtERF1*, *NtERF32*, and *NtERF121* in tobacco (Sears et al., 2014). Therefore, ERF transcription factor expression responds to pathogens and defense-related hormones, such as SA and JA, during stress.

The role of CsERF1 in plant virus infection has not yet been characterized. In this study, we clarified *CsERF1* expression under CTV infection and hormone treatment to determine its function in CTV resistance in sweet orange. Furthermore, the effect of CsERF1 on CTV infection and the expression of key SA and JA signaling pathway genes in *CsERF1*-overexpressing and RNAi-CsERF1 transgenic sweet orange plants were analyzed. The results showed the response of CsERF1 to CTV infection, laying a foundation for further understanding the function of CsERF1 in pathogen infection and the molecular mechanisms of disease resistance in citrus.

## 2 Materials and methods

### 2.1 Plant material

*Nicotiana benthamiana* seedlings were maintained in a growth chamber at 25°C with a 16/8 h light/dark photoperiod. *Agrobacterium tumefaciens* infiltration was conducted on *N. benthamiana* leaves at the four- to six-leaf stage. Two-year-old sweet orange was used as a rootstock to propagate the obtained RNAi-CsERF1 transgenic plants. One-year-old healthy sweet orange seedlings (Madam virons) were treated with exogenous hormones, and plants used in tissue-specific experiments were 5-year-old healthy sweet orange seedlings and sweet orange seedlings infected with CTV, which were stored in the greenhouse of Citrus Research Institute of Southwest University for future use.

### 2.2 Domain analysis and cis-regulatory elements analysis

The conserved domains of the CsERF1 protein were analyzed using the SMART website (http://smart.embl-heidelberg.de/) (Schultz et al., 2000). The promoter of CsERF1 (>Cs5g08360) was downloaded from the Citrus Pan-genome to Breeding Database (CPBD) website (http://citrus.hzau.edu.cn/orange/). The cis-regulatory elements of the CsERF1 gene promoter were analyzed using the PlantCARE database (http://bioinformatics.psb.ugent.be/webtools/plantcare/html/) (Lescot et al., 2002). A comparative analysis of the promoter structure of the CsERF1 gene was performed using TBtools (Toolkit for Biologists integrating various biological data-handling tools) (Chen et al., 2020).

### 2.3 Multiple sequence alignment and Phylogenetic tree construction

The amino acid sequences of *Arabidopsis thaliana* (AtERF1, NP567530.4), *Nicotiana tabacum* (NtERF2, NP001311965.1), *Malus domestica* (MdERF1-like, NP001315734.1), *Vitis vinifera* (VvERF2, RVW50777.1), *Prunus persica* (PpERF1A, XP007209449.1), *Pyrus bretschneideri* (PbERF2-like, XP009369170.1), *Populus euphratica* (PeERF2, XP011031947.1), *Oryza sativa* (OsERF2, XP015623259.1), *Dimocarpus longan* (DIERF1-like, AKE49472.1), *Gossypium hirsutum* (GhERF2, XP016716223.1), *Theobroma cacao* (TcERF2, XP007039732.2) and *Durio zibethinus* (DzERF2-like, XP022718595.1) were searched by BLAST on NCBI website (https://www.ncbi.nlm.nih.gov/). Multiple sequence alignment was performed using DNAMAN software. The protein sequences were used to construct a phylogenetic tree using MEGA 7.0 software with Neighbour-joining method and a bootstrap of 2000 replicates (Kumar et al., 2016).

### 2.4 Subcellular localization of CsERF1 in *N. benthamiana*

Full-length CsERF1 was fused with red fluorescent protein (RFP) at the *BamH* I and *Xho* I sites of the pBI121-RFP vector to obtain pBI121-CsERF1-RFP. The construct was individually transformed into *Agrobacterium tumefaciens* GV3101 and infiltrated into *N. benthamiana*. H2B-GFP was used as nuclear markers. At 48 hours post-inoculation (hpi), fluorescence images of the epidermal cells in infiltrated *N. benthamiana* leaves were obtained using a scanning confocal microscope (FV3000; Olympus).

### 2.5 Hormone treatment

One-year-old healthy sweet orange seedlings were treated with exogenous hormones. 2 mmoL/L SA and 200 μmoL/L MeJA were used as two experimental groups, and ddH_2_O containing 2% ethanol and 0.1% Tween 20 was used as a control group. Each treatment and control group comprised 15 sweet orange seedlings. The optimum condition was uniform wet dripping on the leaf surface. The 3rd–6th leaves from the top of 3 potted seedlings were collected at 0, 3, 6, 12, and 24 h after treatment. The samples were frozen in liquid nitrogen and stored at −80°C until further use.

### 2.6 Citrus transformation

Plant binary expression vector pLGN with a double 35S promoter was used to construct transgenic Wanjincheng orange for overexpressing *CsERF1*. The full-length coding sequence (CDS) of *CsERF1* was amplified from Wanjincheng orange using the primers pLGN-CsERF1-F/R and cloned into pLGN vector at *BamH* I and *EcoR* I sites. A 230 bp fragment of *CsERF1* was amplified using the primers RNAi-CsERF1 230Ff/r and RNAi-CsERF1 230Rf/r by PCR with 5’ sites for *Asc* I and *Swa* I and 3’ sites for *EcoR* I and *BamH* I. The fragments were used as an inverted repeat placed in the RNAi vector puc-RNAi. The primers for vector construction are displayed in **Supplementary Table S1**. The construction was confirmed by sequencing and transformed into *Agrobacterium tumefaciens* EHA105. Citrus transformation was performed as previously described, and confirmed by PCR and β-glucuronidase (GUS) histochemical staining using GUS stain (solarbio) (Zou et al., 2017). Transgenic pLGN-CsERF1 and puc-RNAi-CsERF1 Wanjincheng orange lines were recovered by grafting on 2-year-old virus-free sweet oranges (one to three plants per transgenic line) and maintained in a greenhouse at 23 - 25°C.

### 2.7 Inoculation with CTV

Two-year-old sweet orange was used as a rootstock to propagate the obtained one RNAi-CsERF1 transgenic plant, and eight plants were obtained. Three transgenic and empty vector seedlings were graft inoculated with the CT14A isolate in the greenhouse. The shoots and skins of CT14A sweet orange toxic source were randomly selected, and 2 buds and 1 skin were grafted on each interfered transgenic seedling and empty seedling. The expression of p25 coat protein (CP) gene of CTV was detected using RT-qPCR at 45 days post-inoculation (dpi). The primers used for RT-qPCR are listed in **Supplementary Table S1**.

### 2.8 Total nucleic acid extraction and RT-qPCR analysis

Citrus DNA extraction was performed using the to Biospin Universal Plant Genome DNA Extraction Kit. Expression levels of *CsERF1*, *p25*, *CsPR1*, *NPR1*, *PR5*, *PR1*, *TGA*, *MYC2*, *JAZ* and *LOX2* genes were analyzed by RT-qPCR. Total RNA was extracted from citrus samples using RNAisoPlus (Takara). cDNA was synthesized using All-in-One5×RTMasterMix reverse transcriptase kit (ABM). RT-qPCR was performed using BlasTaq TM 2× qPCR MasterMix premix (ABM). *CsCOX* was used as an internal control gene (Li et al., 2006). Each experiment was performed in triplicate along with the internal control gene. The target gene content was calculated using the 2^−ΔΔCt^ method (Livak and Schmittgen, 2001). The primers used for RT-qPCR are listed in **Supplementary Table S1**.

### 2.9 Statistical analysis

Statistical analysis was performed using GraphPad Prism 8 and data were expressed as mean ± standard deviation (SD) of three biological replicates. Dunnett’s test was used at *p <*0.001 level to assess significant differences in gene expression.

## 3 Results

### 3.1 Expression analysis of CsERF1 gene in different citrus tissues

**Figure 1:**
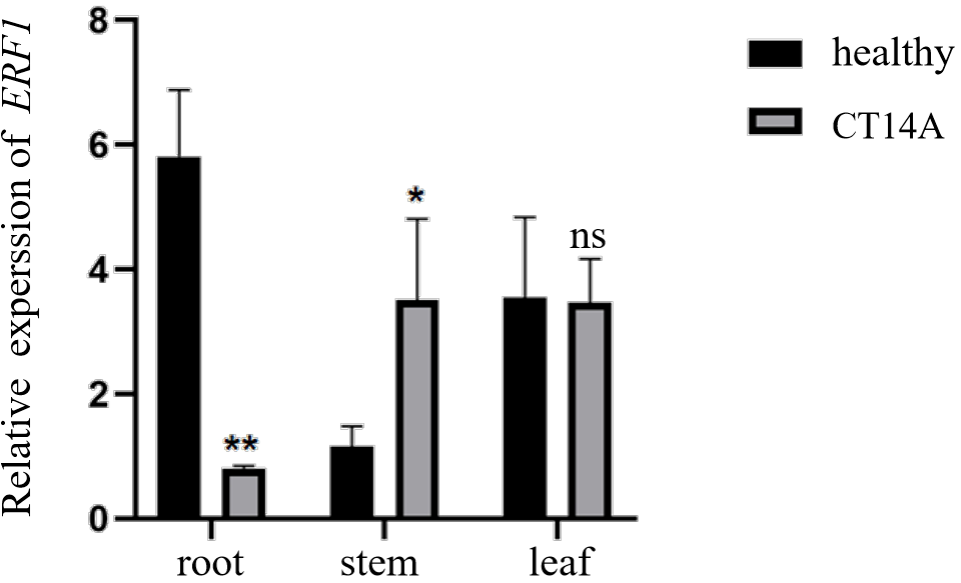
Tissue-specific expression of *CsERF1* in healthy and CTV-infected Citrus. *CsCOX* was used as internal control gene, ns, not significant, *P < 0.05, **P < 0.01, determined by two-way ANOVA using Dunnett’s test.

To explore the potential role of the *CsERF1* gene in biological stress and its expression pattern in different citrus tissues, the total RNA was extracted from roots, stems, and leaves of healthy and CT14A-infected sweet oranges, and the relative expression of the *CsERF1* gene was detected by RT-qPCR. As shown in **Figure 1**, the *CsERF1* gene was expressed in all citrus tissues, but there were differences in expression between tissues. *CsERF1* gene expression in leaves and roots was significantly higher than that in stems. *CsERF1* gene expression decreased significantly in roots and increased significantly in stems under CTV infection, indicating that *CsERF1* gene expression was tissue specific and that the *CsERF1* gene responded to CTV infection, which suggests its regulatory role in CTV stress.

### 3.2 Analysis of domain and cis-regulatory elements of CsERF1

**Figure 2:**
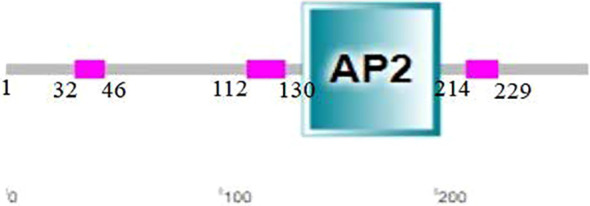
CsERF1 protein structural domain.

**Figure 3:**
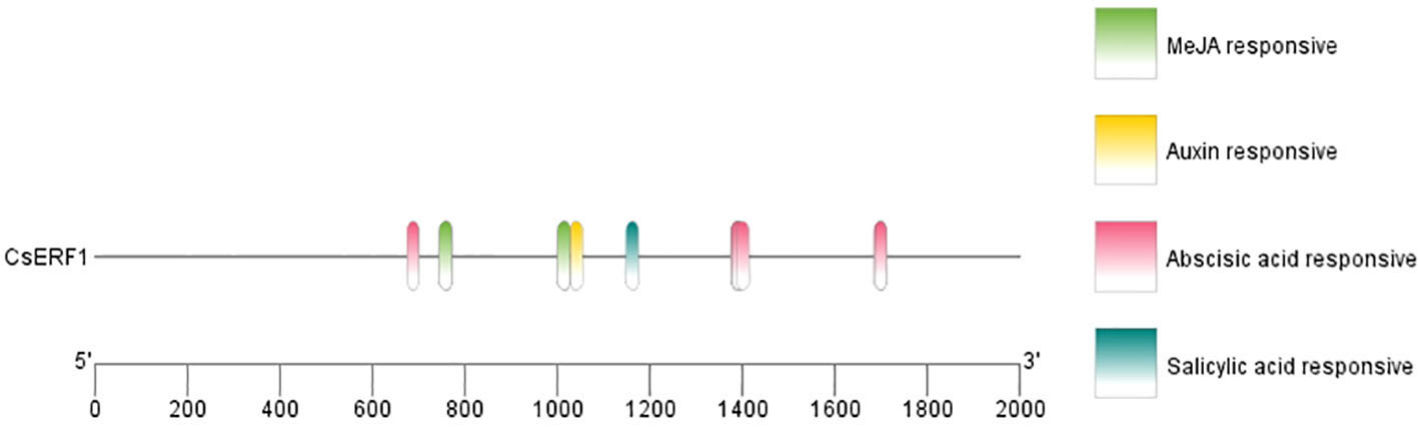
Cis-regulatory element analyses of *CsERF1*. The cis-elements were characterized and indicated by labeling with different colors.

Analysis with SMART revealed that the CsERF1 protein had an AP2 domain located at 138–202 aa (**Figure 2**). To further explore the regulatory mechanism of CsERF1, cis-elements were scanned in the promoter region of CsERF1. A 2000-bp sequence upstream of the translation start was considered a putative promoter region to analyze the distribution of cis-regulatory elements. CsERF1 was mainly regulated by physiological stress (**Supplementary Table S2**). CsERF1 contained by four phytohormone response elements: one SA response-related element, one auxin response-related element, seven ABA response-related elements, and four MeJA response-related elements (**Figure 3**).

### 3.3 Multiple sequence alignment and phylogenetic tree construction of CsERF1

**Figure 4:**
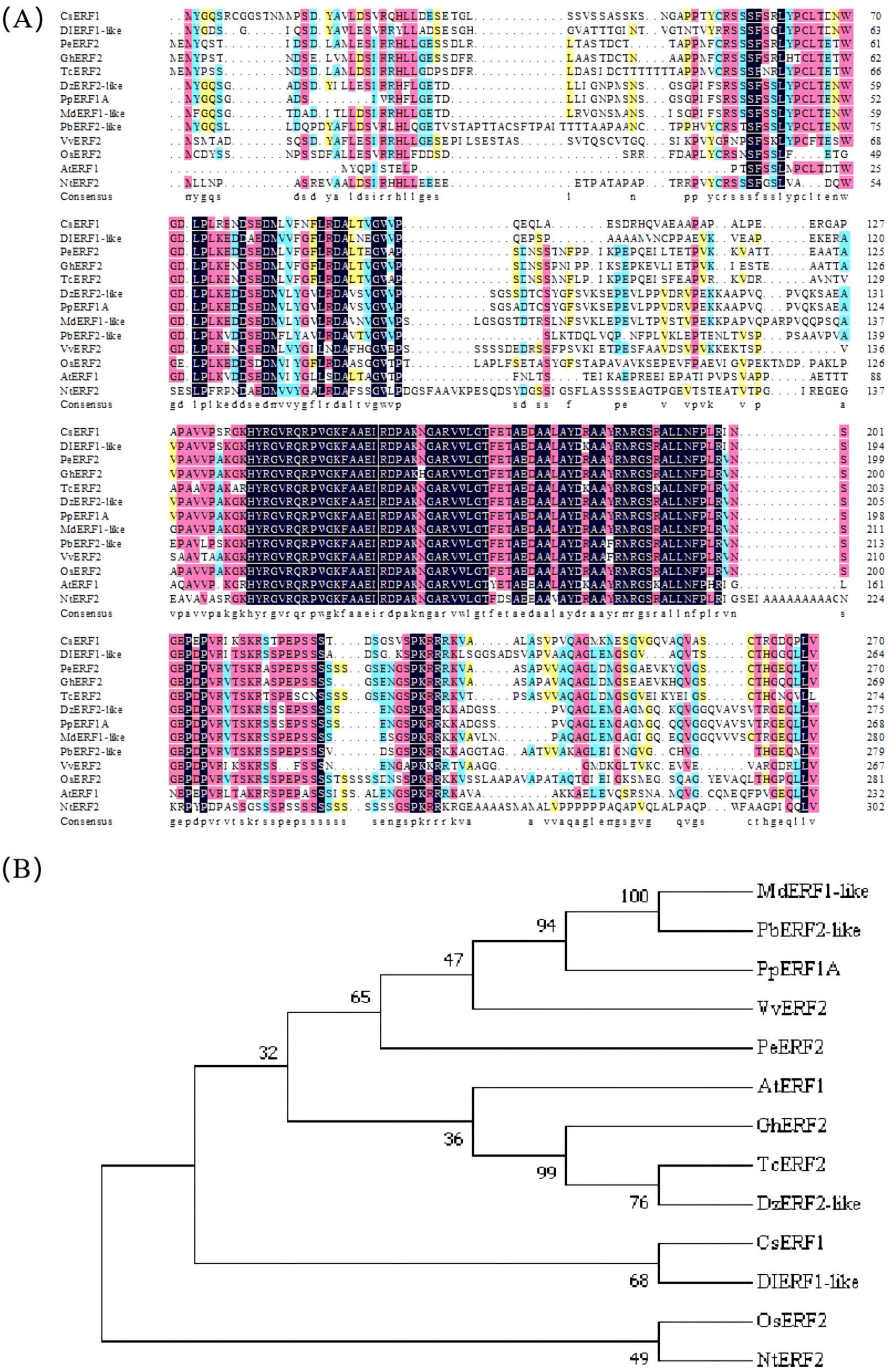
ERF amino acid sequence alignment **(A)** and phylogenetic analysis of different species **(B)**. The phylogenetic tree was generated using the neighbor-joining method and the bootstrap test (2000 replicates). CsERF1, citrus ERF1, Cs5g08360.1; DIERF1-like, *Dimocarpus longan* ERF1-like, AKE49472.1; PeERF2, *Populus euphratica* ERF2, XP011031947.1; GhERF2, *Gossypium hirsutum* ERF2, XP016716223.1; TcERF2, *Theobroma cacao* ERF2, XP007039732.2; DzERF2-like, *Durio zibethinus* ERF2-like, XP022718595.1; PpERF1A, *Prunus persica* ERF1A, XP007209449.1; MdERF1-like, *Malus domestica* ERF1-like, NP001315734.1; PbERF2-like, *Pyrus* x *bretschneideri* ERF2-like, XP009369170.1; VvERF2, *Vitis vinifera* ERF2, RVW50777.1; OsERF2, *Oryza sativa* Japonica Group ERF2, XP015623259.1; AtERF1, *Arabidopsis thaliana* ERF1, NP567530.4; NtERF2, *Nicotiana tabacum* ERF2, NP001311965.1.

To understand the genetic relationship of the CsERF1 protein in different plants, a phylogenetic tree was constructed (**Figure 4**). The amino acid sequence of CsERF1 had relatively high similarity with longan, durian, cacao tree, upland cotton, apple, and pear (64.49, 64.26, 64.1, 61.28, 61.23, and 61.11%, respectively) and relatively lower similarity with peach, poplar, tobacco, *Arabidopsis thaliana*, grape, and rice (59.07, 57.24, 56.44, 54.98, 52.08, and 49.76%, respectively). Phylogenetic tree analysis showed that CsERF1 and DIERF1-like were clustered together and had the closest genetic relationship, suggesting that they had similar functions.

### 3.4 Subcellular localization of CsERF1

**Figure 5:**
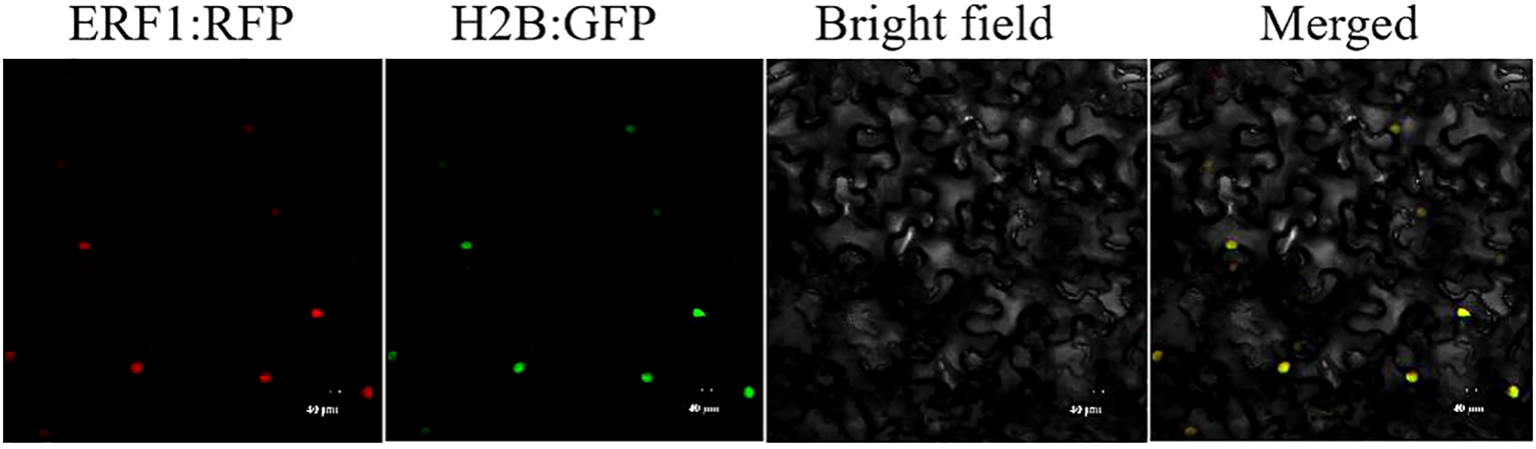
Subcellular localization of CsERF1 in *N. benthamiana* leaf cells. Scale bars represent 40 μm.

In this study, ERF1-RFP and H2B-GFP were transiently expressed in *N.benthamiana* leaves to determine the subcellular localization of CsERF1 protein. Fluorescence observation at 48 hpi showed that ERF1-RFP fusion protein successfully expressed red fluorescence and localized in the nucleus (**Figure 5**).

### 3.5 Expression analysis of *CsERF1* under exogenous SA and MeJA hormone treatment

**Figure 6:**
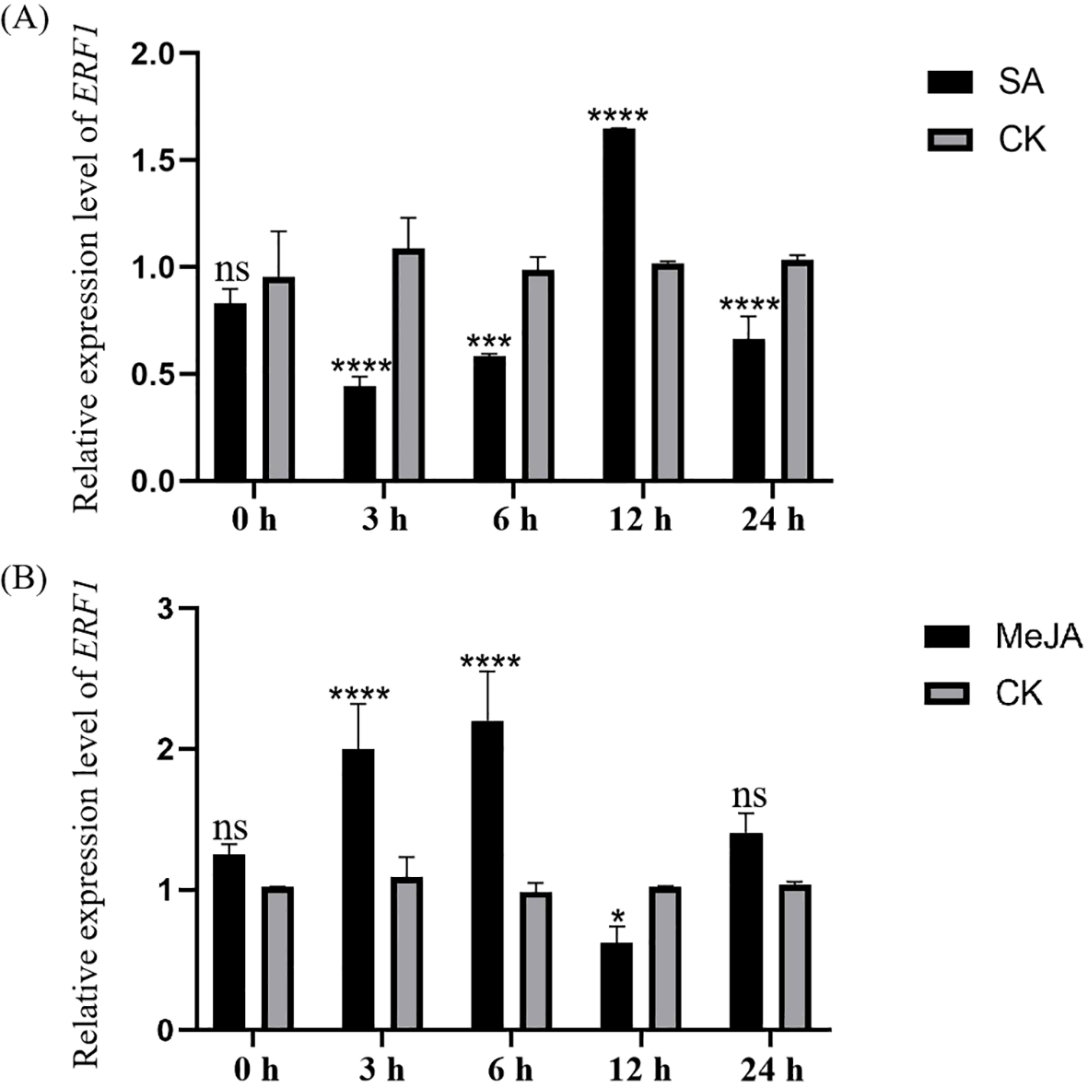
Expression profile of *CsERF1* in sweet orange treated with SA **(A)** and JA **(B)**. *CsCOX* was used as internal control gene, ns, not significant, *P < 0.05, ***P < 0.001, ****P < 0.0001, determined by two-way ANOVA using Dunnett’s test.

After exogenous hormone application, total RNA was extracted from the leaves, and gene expression was analyzed by RT-qPCR. Under exogenous SA treatment, the expression levels of *CsERF1* were 0.41 and 0.59 times higher than that of the control at 3 and 6 hpi, respectively, increasing to a maximum of 1.62 times at 12 hpi and then decreasing to 0.64 times at 24 hpi. Therefore, exogenous SA inhibited *CsERF1* expression at an early stage. Compared to control, the expression level of *CsERF1* was upregulated and then downregulated after exogenous MeJA treatment, reaching a maximum of 2.23-fold at 6 hpi and dropping abruptly to 0.61-fold at 12 hpi (**Figure 6**). Exogenous MeJA inhibited the expression of *CsERF1* at 12 hpi. These results suggest that *CsERF1* plays an important role in responding to hormonal defense signals.

### 3.6 Acquisition of transgenic plants

**Figure 7:**
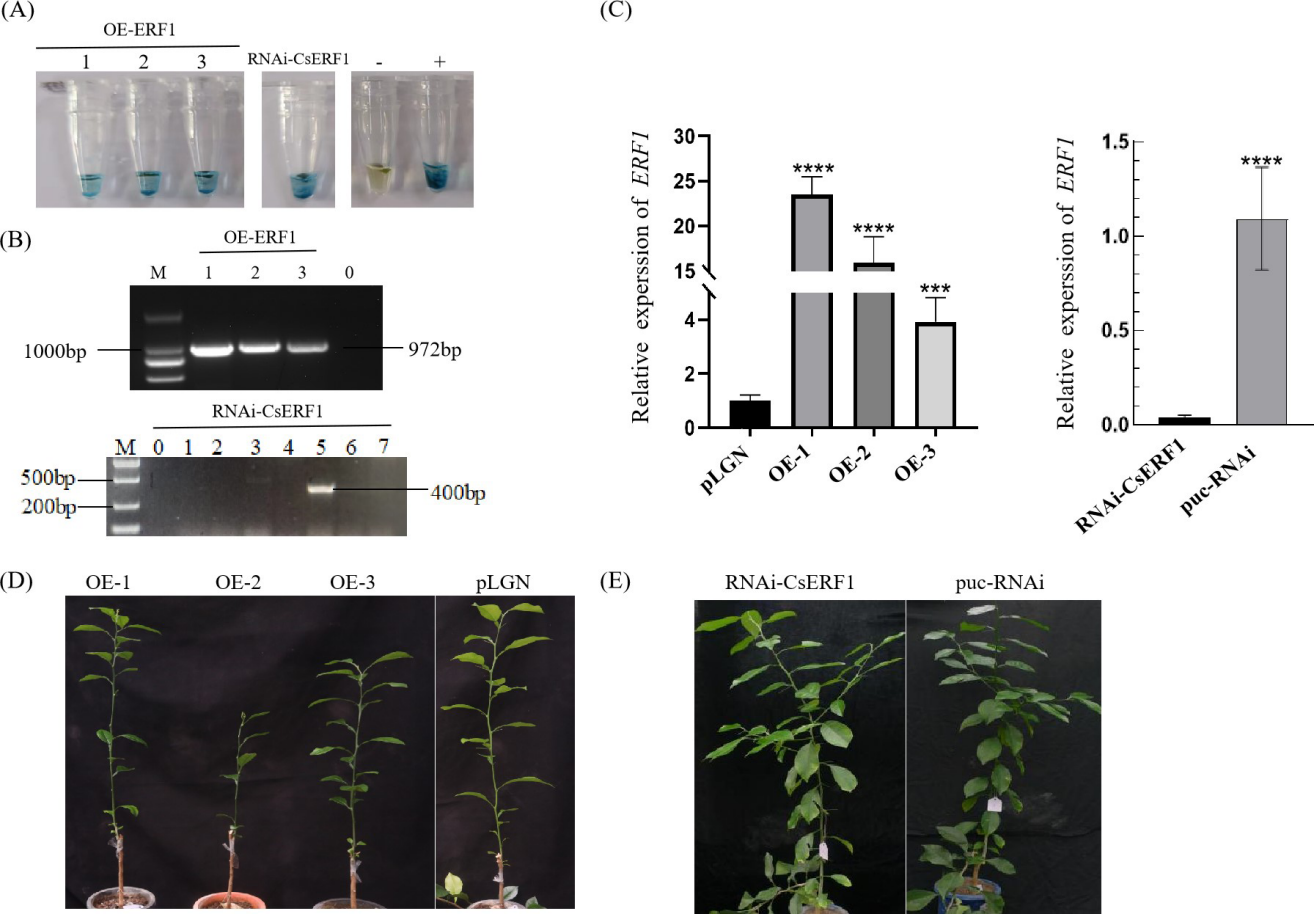
Identification of transgenic plants. **(A)** GUS histochemical staining. **(B)** Detection of the CsERF1 transgenic Wanjincheng orange by PCR, ‘M’ indicates DL 2000 DNA marker. Numbers indicate transgenic lines, ‘0’ represents no-load citrus. **(C)** RT-qPCR detection. **(D)** *CsERF1* overexpression transgenic plants cultured for 180 d in greenhouse. **(E)** RNAi-CsERF1 transgenic plants cultured for 260 d in greenhouse. *CsCOX* was used as internal control gene, ***P < 0.001, ****P < 0.0001, determined by one-way ANOVA with Dunnett’s test.

The interference expression vector puc-RNAi-CsERF1 and the overexpression vector pLGN-CsERF1 were transformed into the Wanjincheng (*Citrus sinensis*) via *Agrobacterium*-mediated epicotyl transformation. Three independent *CsERF1* overexpression transgenic lines (OE1-3) and one independent *CsERF1* interference transgenic line were detected by GUS histochemical staining, PCR and RT-qPCR identification. In addition, no significant phenotypic differences were observed between transgenic *CsERF1* and wild-type (WT) sweet orange during the observation period of 6 months to 1 year (**Figure 7**).

### 3.7 Effect of RNAi-CsERF1 transgenic plants on CTV infection

**Figure 8:**
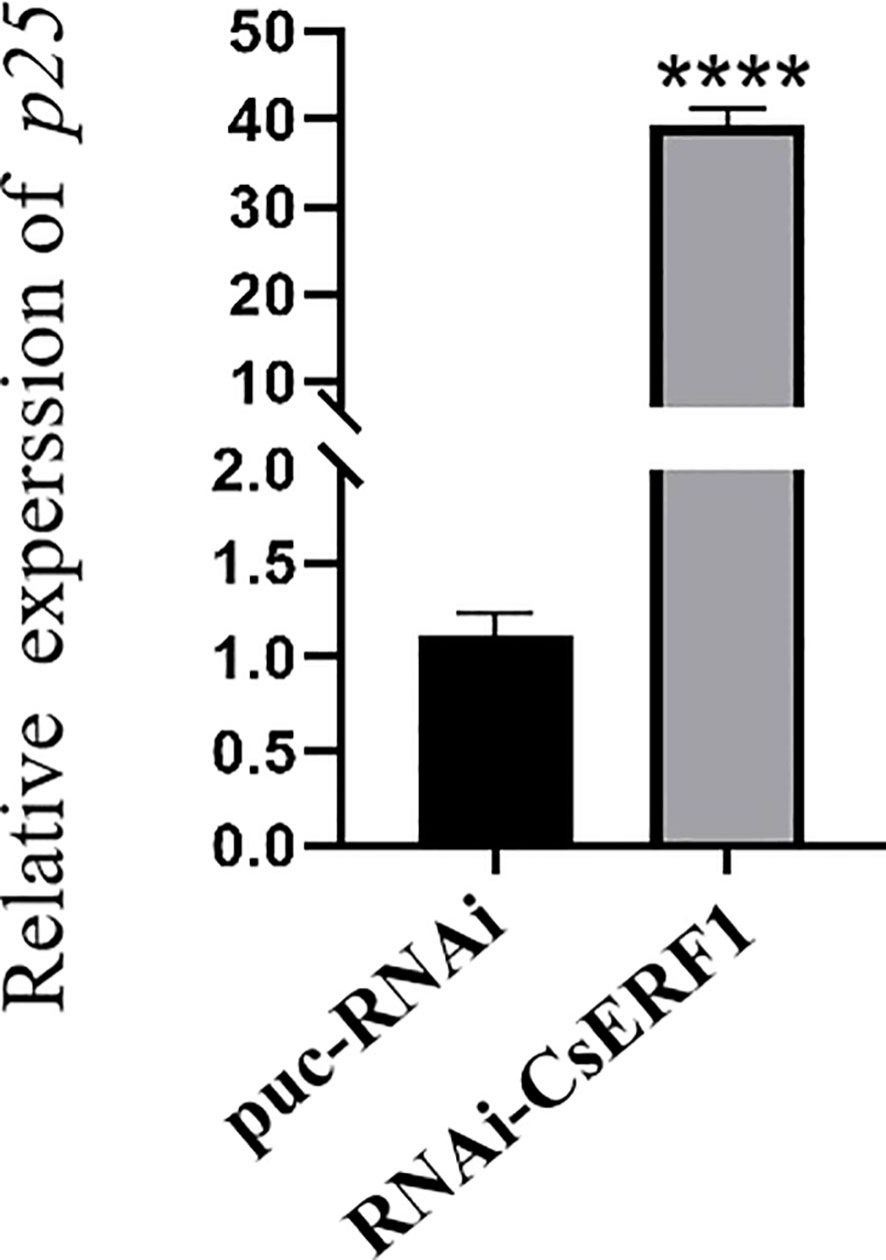
RNAi-CsERF1 transgenic plants promoted the infection of CTV. *CsCOX* was chosen as internal control gene, data analysis was determined by two-way ANOVA using Dunnett’s test, ****P < 0.0001.

The effect of RNAi-CsERF1 transgenic plants on CTV infection was evaluated by determining the expression levels of p25 in plants using RT-qPCR. At 45 dpi, CTV was detected in both transgenic and control plants, but *p25* expression levels were significantly higher in RNAi-CsERF1 transgenic plants than in control plants (**Figure 8**). Therefore, CsERF1 interference promoted CTV infection.

### 3.8 Expression of JA and SA signaling pathway genes in CsERF1 transgenic plants

**Figure 9:**
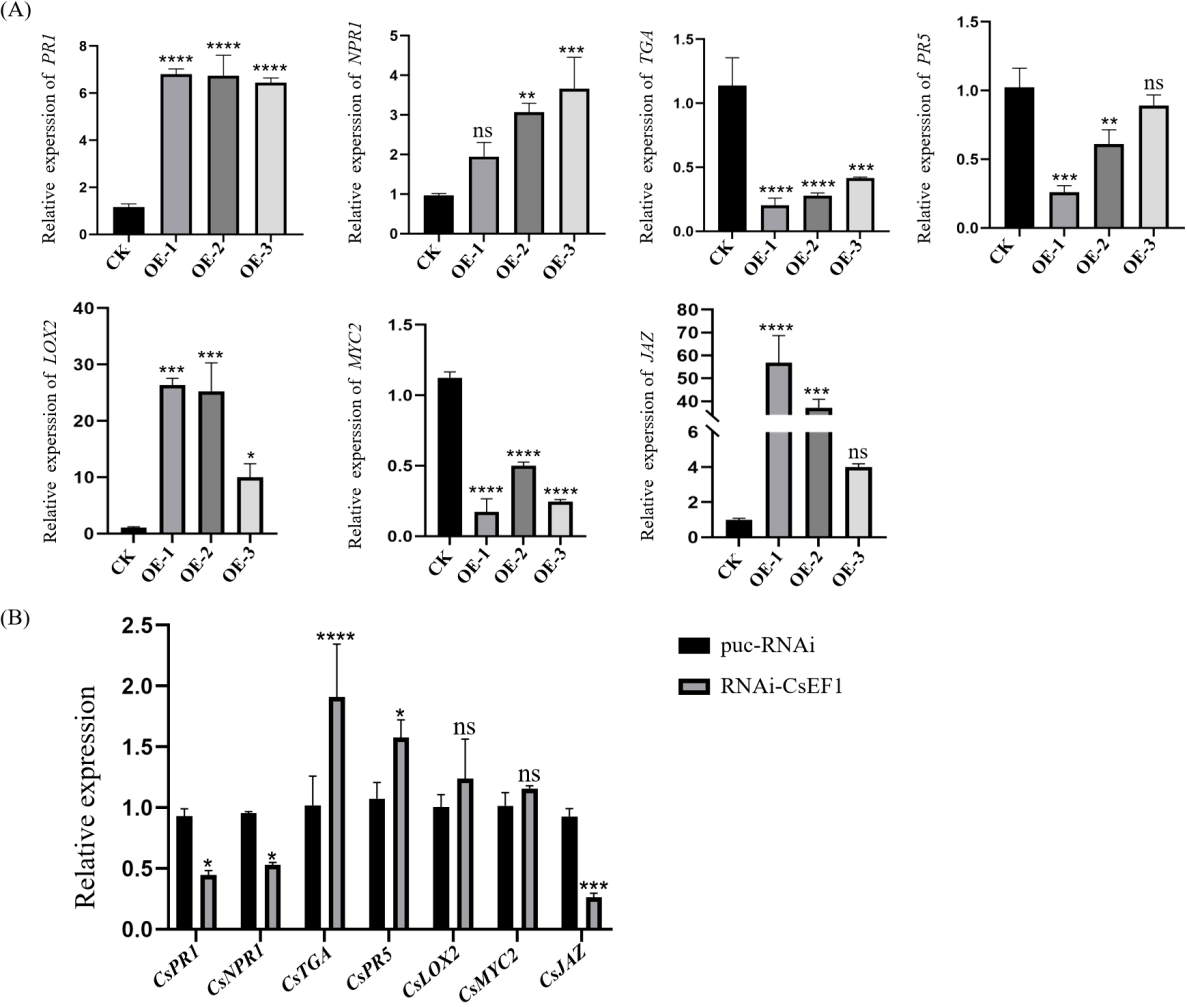
Expression of defense-related genes in transgenic citrus plants. **(A)** Expression of genes related to SA and JA signaling pathways in *CsERF1*-overexpressing citrus plants. **(B)** Expression of genes related to SA and JA signaling pathway in RNAi-CsERF1 citrus plant. ns, not significant, *P < 0.05, **P < 0.01, ***P < 0.001, ****P < 0.0001, determined by two-way ANOVA and Dunnett’s test.

To further investigate the effect of CsERF1 on SA and JA signaling pathway-related genes, we employed RT-qPCR to investigate transcriptional changes in *NPR1*, *PR5*, *PR1*, and *TGA* in SA pathways and *MYC2*, *JAZ*, and *LOX2* in JA pathways in CsERF1-overexpressing and RNAi-CsERF1 transgenic plants. *NPR1*, a positive regulator of the SA pathway (Chen et al., 2013), and *PR1*, a activator gene of SAR (Fu and Dong, 2013), were upregulated in overexpressing transgenic citrus, indicating that overexpressing *CsERF1* activated the SA defense pathway (**Figure 9A**). The JA synthesis gene *LOX2* was upregulated (Wasternack and Hause, 2013). *MYC2*, a positive regulatory element of JA signaling (Dombrecht et al., 2007), was downregulated, and *JAZ*, an inhibitor of the JA pathway (Thines et al., 2007), was upregulated, indicating that *CsERF1* overexpression inhibited the JA defense pathway (**Figure 9B**), consistent with the results showing that CsERF1 expression was antagonized by SA and JA in the exogenous hormone application experiment.

## 4 Discussion

At present, the function of AP2/ERF transcription factors is an important research topic. ERF genes have been widely studied in model plants, but the gene function of the AP2/ERF gene family in sweet orange is relatively less studied. The AP2/ERF superfamily is an important transcription factor group involved in plant growth,development and stress responses (Ma et al., 2024). Zhang found that the expression levels of *MdERF100* in apples were significantly higher in roots and leaves than in stems (Zhang, 2022). Consistent with these results, the results of the present study showed that the expression patterns of the *CsERF1* gene differed in citrus roots, stems, and leaves and that the expression level of this gene was higher in roots and leaves than in stems. The expression level of *CsERF1* gene was significantly reduced in the roots of citrus and was significantly increased in the stems after CTV infection. As we know, CTV is a phloem-restricted virus, indicating that CsERF1 can respond to CTV stress. The subcellular localization of proteins plays an important role in revealing their biological functions. Most transcription factors are localized in the nucleus to regulate the transcription of downstream genes, thus realizing their biological functions (Mei et al., 2018). This study also found that CsERF1 transcription factor is localized in the nucleus, and CsERF1 has high homology (64.49%) with ERF1-like amino acid sequence from longan.

Many studies have shown that the expression levels of the ERF family change after treatment with pathogens and exogenous hormones. An ERF gene *CaPTI1* was induced by *Phytophthora capsici*, SA, MeJA and ethephon (ETH), and the expression of *CaERF5* gene was up-regulated (Nie and Wang, 2023). *Arabidopsis thaliana At4g13040* is a member of the AP2/ERF family. *At4g13040* is a positive regulator of disease defense that functions upstream of SA accumulation. The gene is upregulated in pathogen inoculation and exogenous SA treatment (Giri et al., 2014). In the present study, *CsERF1* expression was inhibited by exogenous SA but induced by exogenous MeJA in the early stage of hormone treatment, suggesting that CsERF1 plays an important role in the response to hormone signal transduction.

Regulation of plant defense responses is a complex process. Many defense responses depend on SA and JA pathways, and there is antagonism between SA and JA pathways.The expression of *HSP17.4* activated the up-regulation of downstream signals of SA and inhibited the JA signal pathway in strawberry. Moreover, the *HSP17.4* gene-silenced cultivar Sweet Charlie plants were more susceptible to *Colletotrichum gloeosporioides* than the wild-type Sweet Charlie. This suggests that *HSP17.4* mediates SA and JA pathways in the regulation of resistance to *Colletotrichum gloeosporioides* in strawberry (Fang et al., 2021). *CAT2* plays an important role in plant defense against pathogen attack by regulating antagonistic signaling pathways between SA and JA (Yuan et al., 2017). ERF overexpression in plants has been shown to alter defense gene expression and pathogen resistance (Guo et al., 2004; Zhang et al., 2004). Overexpression of *MdERF11* gene in apple and *AcERF2* gene in *Atriplex canescens* activates defense responses and enhances disease resistance by increasing the expression of genes related to the SA defense pathway (Sun et al., 2018; Wang et al., 2020). In wheat, *TaERF3* is mainly involved in the active defense response to *Blumeria graminis* at an earlier stage through SA signaling (Zhang et al., 2007). Silencing *CaAP2/ERF064* compromised pepper plant resistance to *Phytophthora capsici*, while the ectopic expression of *CaAP2/ERF064* in *N*. *benthamiana* plants elevated the expression level of *NbPR1b*, *NbPR3*, and *NbPR4* and enhanced resistance to *P*. *capsici* (Jin et al., 2019). The expression of PR genes, such as *PR1*, *PR2*, *PR4*, Osmotin and *SAR8.2*, were activated and resistance to tobacco mosaic virus (TMV), *Ralstonia solanacearum* and *Alternaria alternata* was enhanced in *GmERF3* gene-overexpressing transgenic tobacco plants (Zhang et al., 2009). In the present study, the *CsERF1* overexpression and RNAi-CsERF1 transgenic sweet orange were analyzed using RT-qPCR. *PR1* and *NPR1* were upregulated, while the JA signal positive regulatory element *MYC2* was downregulated and the inhibitor JAZ was upregulated in *CsERF1* overexpressing transgenic sweet orange transgenic plants. This evidence suggests that *CsERF1* overexpression activated the SA defense pathway and inhibited the JA defense pathway, consistent with the results of the hormone experiments. *CsERF1* expression was antagonized by SA and JA. Moreover, CTV titers in RNAi-CsERF1 transgenic sweet orange plants were significantly higher than those in control plants. Suggesting that interference with CsERF1 can promote the CTV infection. This study indicated that CsERF1 had great potential in plant virus resistance, and provided reference for breeding resistant varieties. In the future, we can explore whether there are proteins interacting with CsERF1 in plants, and further understand the biological function of CsERF1.

## 5 Conclusion

The citrus transcription factor CsERF1 is involved in the response to citrus tristeza disease. Interfering with *CsERF1* expression enhanced the susceptibility of sweet orange to CTV. *CsERF1* responded to exogenous SA and MeJA hormone treatment. Expression analysis of genes related to SA and JA defense signaling pathways in transgenic plants suggested that CsERF1 is involved in SA and JA-mediated defense pathways, regulating the expression of key genes, such as PR, to stimulate the citrus immune response to CTV infection. The results of this study indicate the potential of CsERF1 in plant virus resistance and provide a reference for breeding excellent resistant varieties.

## Data Availability

The data presented in the study are deposited in the NCBI (https://www.ncbi.nlm.nih.gov/) repository. *Arabidopsis thaliana* (AtERF1, NP567530.4), *Nicotiana tabacum* (NtERF2, NP001311965.1), *Malus domestica* (MdERF1-like, NP001315734.1), *Vitis vinifera* (VvERF2, RVW50777.1), *Prunus persica* (PpERF1A, XP007209449.1), *Pyrus bretschneideri* (PbERF2-like, XP009369170.1), *Populus euphratica* (PeERF2, XP011031947.1), *Oryza sativa* (OsERF2, XP015623259.1), *Dimocarpus longan* (DIERF1-like, AKE49472.1), *Gossypium hirsutum* (GhERF2, XP016716223.1), *Theobroma cacao* (TcERF2, XP007039732.2) and *Durio zibethinus* (DzERF2-like, XP022718595.1).
