# Supplementary material for: Citrus transcription factor CsERF1 is involved in the response to citrus tristeza disease
Source: Front Plant Sci. 2025 Jan 14;15:1528348. doi: 10.3389/fpls.2024.1528348 (PMC11772405; doi:10.3389/fpls.2024.1528348)
Supplement: Supplementary file 1 [file Table1.docx]

**Table S1** Primers designed in experiment

| Primer name | Primer sequence （5’-3’） |
| --- | --- |
| pBI121-CsERF1-RFP-F | AAGATGCCTCTGCCGACA |
| pBI121-CsERF1-RFP-R | ATGTCCCAGGCGAAGG |
| pLGN-CsERF1-F | GGACAGGGTACCCGGGGATCCATGGATTACAAGGATGATGATGATAAG |
| pLGN-CsERF1-R | TCTCATTAAAGCAGGGAATTCTCAGCTTACCAATGGCTGGTC |
| RNAi-CsERF1230 Ff | TTGGCGCGCCCCCTAAGGATCAACTCGGGTG |
| RNAi-CsERF1230 Fr | GCGATTTAAATTCAGCTTACCAATGGCTGGTC |
| RNAi-CsERF1230 Rf | CGGGAATTCCCCTAAGGATCAACTCGGGTG |
| RNAi-CsERF1230 Rr | CGCGGATCCTCAGCTTACCAATGGCTGGTC |
| RT-CsNPR1 F | AGAGGACCCAAGTTTGAGCT |
| RT-CsNPR1 R | CAGGCATCATCATCCACAC |
| RT-CsTGA F | TGACAAAGAACTTCAGAAAGG |
| RT-CsTGA R | CGTCGGTTCCACATCATAG |
| RT-CsPR1 F | AAATGTGGGTGAATGAGAAAGC |
| RT-CsPR1 R | ATTATTGTTGCACGTCACCTTG |
| RT-CsPR5 F | CACCATTGCCAATAACCCTAATG |
| RT-CsPR5 R | GGGACAGTTACCGTTAAGATCAG |
| RT-LOX2 F | TGCCAGGTTTGCTAAGACAC |
| RT-LOX2 R | GGCCATTCCGTAATCAACTG |
| RT-MYC2 F | CATCAACAGATTCCGCAGACAC |
| RT-MYC2 R | CCGCAAAATTCAATATCTCCCC |
| RT-JAZ F | TCGAGCTACCGTTGAACTC |
| RT-JAZ R | GCCTTGAATGTCTCGAAAGC |
| RT P25-q2F | GGACTTTCACTTAGCTATG |
| RT P25-q2R | CGTTACTTCTACCCCAGAC |
| RT-CsERF1-q3F | CACGGATAGTGGCAGTGTGT |
| RT-CsERF1-q3R | TTCATCCCGGCTTGTACTGG |
| COXf | GTATGCCACGTCGCATTCCAGA |
| COXr | GCCAAAACTGCTAAGGGCATTC |
| pBI121-CsERF1-RFP-F | AAGATGCCTCTGCCGACA |
| pBI121-CsERF1-RFP-R | ATGTCCCAGGCGAAGG |
