# Supplementary material for: Citrus transcription factor CsERF1 is involved in the response to citrus tristeza disease
Source: Front Plant Sci. 2025 Jan 14;15:1528348. doi: 10.3389/fpls.2024.1528348 (PMC11772405; doi:10.3389/fpls.2024.1528348)
Supplement: Supplementary file 2 [file Table2.docx]

**Table S2** *CsERF1* promoter component statistics

| Cis-regulatory element | sequence | quantity | name |
| --- | --- | --- | --- |
| ARE | AAACCA | 4 | Anaerobic induction |
| TGACG-motif | TGACG | 2 | MeJA responsive |
| CGTCA-motif | CGTCA | 2 | MeJA responsive |
| G-Box | CACGTC，CACGTT，CACGAC，CACGTG | 8 | Light responsive |
| TATA-box | ATATAA，TATA，ATATAT，TATATA，ATTATA，TATAA，TACAAAA，ccTATAAAaa，TATAAAA，TATAAA，TACATAAA |  | core promoter element of transcription start |
| MRE | AACCTAA | 1 | Light responsive |
| ABRE | ACGTG，CACGTG，CGCACGTGTC | 7 | Abscisic acid responsive |
| Box 4 | ATTAAT | 2 | Light responsive |
| CCAAT-box | CAACGG | 2 | Light responsive |
| GATA-motif | AAGGATAAGG | 2 | Light responsive |
| GT1-motif | GGTTAA | 1 | Light responsive |
| LAMP-element | CCTTATCCA，CTTATCCA | 2 | Light responsive |
| TCT-motif | TCTTAC | 3 | Light responsive |
| MBS | CAACTG | 2 | Light responsive |
| LTR | CCGAAA | 1 | Low-temperature responsive |
| I-Box | atGATAAGGTC | 1 | Light responsive |
| CAT-box | GCCACT | 2 | Meristem expression |
| ATCT-motif | AATCTAATCC | 1 | Light responsive |
| AT-rich element | ATAGAAATCAA | 2 | binding site of DNA binding protein |
| GCN4-motif | TGAGTCA | 1 | endosperm expression |
| RY-element | CATGCATG | 1 | seed-specific regulation |
| TCA-element | CCATCTTTTT | 1 | Salicylic acid responsive |
| TGA-element | AACGAC | 1 | Auxin responsive |
